# Nucleolar and spindle associated protein 1 enhances chemoresistance through DNA damage repair pathway in chronic lymphocytic leukemia by binding with RAD51

**DOI:** 10.1038/s41419-021-04368-2

**Published:** 2021-11-15

**Authors:** Yang Han, Xinting Hu, Xiaoya Yun, Jiarui Liu, Juan Yang, Zheng Tian, Xin Zhang, Ya Zhang, Xin Wang

**Affiliations:** 1 Department of Hematology, Shandong Provincial Hospital, Cheeloo College of Medicine, Shandong University, Jinan, Shandong 250021 China; 2 Department of Hematology, Shandong Provincial Hospital Affiliated to Shandong First Medical University, Jinan, Shandong 250021 China; 3 School of Medicine, Shandong University, Jinan, Shandong 250012 China; 4 Shandong Provincial Engineering Research Center of Lymphoma, Jinan, Shandong 250021 China; 5 Branch of National Clinical Research Center for Hematologic Diseases, Jinan, Shandong 250021 China; 6 National Clinical Research Center for Hematologic Diseases, the First Affiliated Hospital of Soochow University, Suzhou, 251006 China

**Keywords:** Oncogenes, Oncogenesis

## Abstract

Nucleolar and spindle-associated protein 1 (NUSAP1) is an essential regulator of mitotic progression, spindle assembly, and chromosome attachment. Although NUSAP1 acts as an oncogene involved in the progression of several cancers, the exact role of chronic lymphocytic leukemia (CLL) remains elusive. Herein, we first discovered obvious overexpression of NUSAP1 in CLL associated with poor prognosis. Next, the NUSAP1 level was modulated by transfecting CLL cells with lentivirus. Silencing NUSAP1 inhibited the cell proliferation, promoted cell apoptosis and G0/G1 phase arrest. Mechanistically, high expression of NUSAP1 strengthened DNA damage repairing with RAD51 engagement. Our results also indicated that NUSAP1 knockdown suppressed the growth CLL cells in vivo. We further confirmed that NUSAP1 reduction enhanced the sensitivity of CLL cells to fludarabine or ibrutinib. Overall, our research investigates the mechanism by which NUSAP1 enhances chemoresistance via DNA damage repair (DDR) signaling by stabilizing RAD51 in CLL cells. Hence, NUSAP1 may be expected to be a perspective target for the treatment of CLL with chemotherapy resistance.

## Introduction

Chronic lymphocytic leukemia (CLL) is the most prevalent leukemia among the elderly in western countries featured by a heterogeneous clinical course and easy recurrence [1]. In recent years, many genetic abnormalities are associated with refractory course, poor prognosis, and chemoresistance [2]. Additionally, DNA damage is a recurring phenomenon and serves as a major factor in cancer development inducing normal cells to obtain oncogenic mutations [3]. Of note, suppression of the DNA repair process is considered to be a promising approach for CLL treatment [4].

Nucleolar and spindle-associated protein 1 (NUSAP1), with a molecular weight of 55KD, serves as a microtubule-binding protein in chromosome separation, spindle assembly, and plays significant role to ensure normal regulation of cell cycle as well [5]. Previous studies suggested that NUSAP1 was abnormally elevated in numerous tumors including liver cancer [6], breast cancer [7], prostate cancer [8], gastric cancer [9], and bladder cancer [10]. High expression of NUSAP1 correlates with the adverse prognosis of cervical cancer [11] and breast cancer [7]. In addition, NUSAP1 engages in the biological process such as cell proliferation, apoptosis, cell cycle and metastasis in several types of cancers by regulating Wnt/β-catenin [12], Hedgehog [13], PI3K/AKT [14], Hippo-Yap1 [9], and other pathways. It was reported that NUSAP1 participated in the cellular DNA damage response process [15]. However, its function and mechanism in the development of CLL remain elusive.

RAD51 protein is involved in DNA damage, cell cycle, apoptosis regulation [16], and chemotherapy resistance [17]. Elevated levels of RAD51 were uncovered in a variety of tumors, including cervical cancer [18], nonsmall cell lung cancer [19], breast cancer [20], ovarian cancer [21], and pancreatic cancer [22]. On basis of previous analysis that NUSAP1 was associated with homologous recombinant (HR) belonged to DNA repair process, we hypothesized whether NUSAP1 influenced DNA repairing via interacting with RAD51, the most important enzyme catalyzing HR [23].

Herein, we aimed first to investigate the expression pattern and functional consequences of NUSAP1 in CLL development. Additionally, the underlying mechanism of NUSAP1 in relation to the DNA damage process was characterized to elucidate the possibility of induction of chemoresistance. Remarkably, excessive NUSAP1 expression was displayed with adverse prognosis in CLL specimens and cells. Collectively, our results revealed that NUSAP1 served as an oncogene leading to activate DNA damage repair pathway by binding with RAD51, which providing theoretical basis for novel molecular markers and targeted therapy in CLL.

## Results

### Overexpression of NUSAP1 in CLL specimens and cell lines

**Fig. 1:**
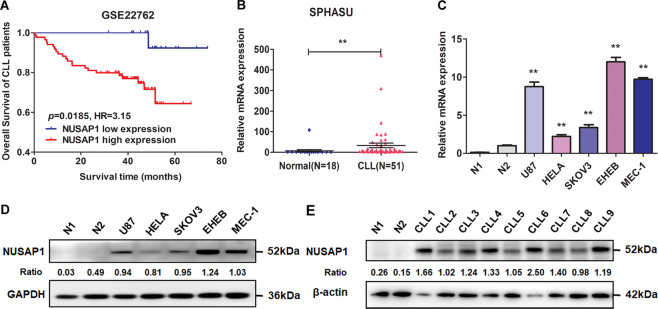
NUSAP1 was elevated in CLL and related to prognosis of CLL patients. **A** Overall survival (OS) curves of CLL patients based on GSE22762 with stratified NUSAP1 expression. **B** High mRNA expression of NUSAP1 was presented in CLL primary cells. **C** Excessive mRNA expression of NUSAP1 was detected in CLL cell lines (MEC-1, EHEB) than in both normal CD19 + B cells and other cancer cells by qRT-PCR. **D** High protein expression of NUSAP1 was observed in CLL cell lines. **E** High protein expression of NUSAP1 was observed in CLL patients than normal CD19 + B cells. Data are shown as the mean ± SD, *n* = 3. *p < 0.05; **p < 0.01.

To explore the role of NUSAP1, the overall survival (OS) analyzing through Kaplan–Meier method in CLL patients was employed. The NUSAP1 relative expression values above 5.39 were defined as high expression. According to the statistical analysis database GSE22762, the results showed that patients with high expression of NUSAP1 had significantly shorter OS than those with low expression (Fig. 1A). Meanwhile, CLL specimens were also chosen for qRT-PCR analysis (Fig. 1B). The expression level of NUSAP1 was obviously elevated in CLL specimens than in the normal group, indicating that patients with NUSAP1 rising acquired a poor prognosis. We determined the cut-off value according to 50% CLL patients with NUSAP1 relative expression values above 6.61. The high expression of NUSAP1 was statistically correlated with increased WBC count (≥40*10^9^/L; *p* = 0.003), Binet stage (C, *p* = 0.035), Rai stage (III/IV; *p* = 0.003), β2-microglobulin (≥3 mg/L; *p* = 0.036), IGHV mutation (*p* = 0.043), and del(11q)/del(17q)/Tp53 mutation (*p* = 0.038) (Table 1). Moreover, mRNA and protein expression levels in the MEC-1 and EHEB cell lines were detected noticeably higher than that in both normal CD19 + B cells of healthy donors and other cancer cells such as U87 (glioblastoma cell line), HELA (cervical cancer cell line), SKOV3 (ovarian cancer cell line) cells (*p* < 0.001) (Fig. 1C, D). The protein expression levels of NUSAP1 in CLL specimens were generally higher than normal CD19 + B cells (Fig. 1E). It was speculated that NUSAP1 protein may be involved in the oncogenic process in CLL.

**Table 1:** Correlation between NUSAP1 expression and characteristics of CLL patients (*n* = 51).

Characteristics	NUSAP1 expression		*p* value
	Low	High	
No. of patients	25	26	
Gender			
Male	18	15	0.285
Female	7	11	
Age (years)			
≥60	14	16	0.688
<60	11	10	
WBC (×10^9^/L)			
≥40	6	17	0.003**
<40	19	9	
Binet stage			
A/B	17	10	0.035*
C	8	16	
Rai stage			
0/I/II	18	8	0.003**
III/IV	7	18	
LDH (U/L)			
≥222	8	14	0.074
<222	17	12	
β2-MG (mg/L)			
≥3	9	17	0.036*
<3	16	9	
CD38			
Positive	3	3	0.959
Negative	22	23	
ZAP-70			
Positive	1	3	0.276
Negative	23	20	
IGHV status			
Mutated	15	8	0.043*
Unmutated	9	16	
FISH			
Normal, del(13q) or trisomy 12	17	11	0.038*
del(11q), del(17p) or Tp53 mutation	3	9	

*LDH* lactate dehydrogenase, *ZAP-70* 70-KD zeta-associated protein, *IGHV* immunoglobin heavy chain variable region, *FISH* fluorescence in situ hybridization.

Bold values identify statistical significance (*p* < 0.05)

*p < 0.5; **p < 0.01

### Functional enrichment analyses of NUSAP1 through RNA-sequencing in CLL cells

**Fig. 2:**
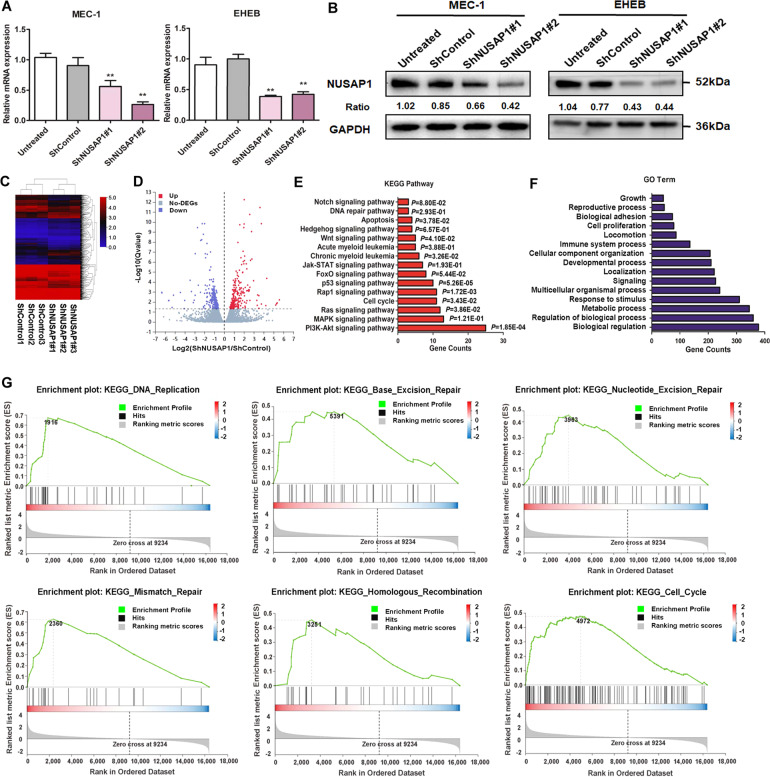
RNA-seq analysis of NUSAP1 between ShControl and ShNUSAP1 cells. **A** Efficiency verification of NUSAP1 knockdown in MEC-1 cells by qRT-PCR. **B** Efficiency verification of NUSAP1 knockdown in MEC-1 cells by western blot. **C** The heatmap of different gene-expression in MEC-1 after lentivirus transfection. **D** Gene volcano map about 277 down and 236 upregulated genes. **E** KEGG pathway analysis of NUSAP1 expression. **F** GO terms analysis of differently expressing genes. (**G**) GSEA analysis of different gene expression correlated with NUSAP1 was performed. NES normalized enrichment score. Data are shown as the mean ± SD, *n* = 3. *p < 0.05; **p < 0.01.

To figure out the features of NUSAP1, we designed two short hairpin RNAs (shRNAs) to decrease the expression of NUSAP1 in MEC-1 and EHEB cells (Fig. 2A, B). RNA-sequencing was implemented between the MEC-1 cells transfected ShControl and ShNUSAP1#2 respectively (Fig. 2C). Totally, 236 upregulated genes and 277 downregulated genes were screened for subsequent analyses (Fig. 2D). As illustrated in Fig. 2E, it has been revealed that NUSAP1 were enriched in pathways related to cancer, such as DNA repair pathway, PI3K-AKT signaling pathway, and p53 signaling pathway through analysis of kyoto encyclopedia of genes and genomes (KEGG). Gene ontology (GO) analysis implicated that NUSAP1 was closely associated with biological processes including cell proliferation, cell cycle, and response to stimulus (Fig. 2F). Gene set enrichment analysis (GSEA) indicated that NUSAP1 was mainly enriched in DNA replication, homologous recombination, base excision repair, nucleotide excision repair, mismatch repair, and cell cycle (Fig. 2G). In summary, it was analyzed NUSAP1 potentially promoted CLL occurrence through managing several oncogenic signaling pathways, especially in the DNA repair pathway.

### Silencing of NUSAP1 decreased cell proliferation and enhanced apoptosis accompanied with cell cycle arrest in CLL cells

**Fig. 3:**
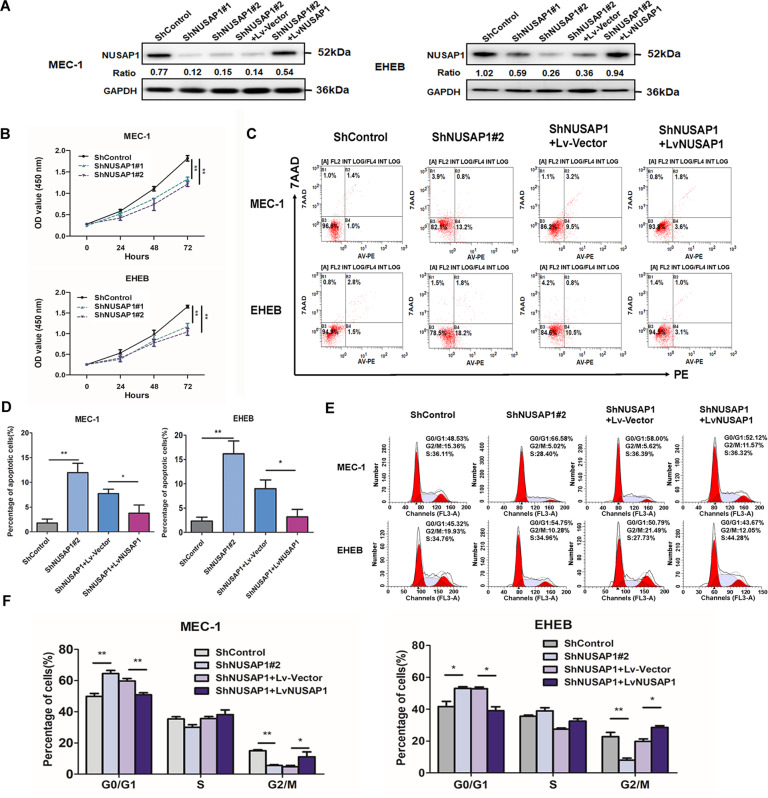
Cell proliferation decline, apoptosis rising and cell cycle arrest accompanied with NUSAP1 silencing. **A** Western blotting verification of CLL cells with NUSAP1 knockdown and recovered. **B** OD values of NUSAP1 knockdown CLL cells by CCK-8. **C** Test of apoptosis by flow cytometry in cells with NUSAP1 decrement and recovered. **D** The relative apoptotic rates in CLL cells with NUSAP1 knockdown and recovered. **E** Detection of cell cycle in cells with NUSAP1 decrement and recovered by flow cytometry assay. **F** The relative rates of cells in different cell cycle phases. All data are presented as the mean ± SD, *n* = 3. *p < 0.05; **p < 0.01.

The high expression of NUSAP1 in CLL suggested that NUSAP1 might serve as a factor in promoting CLL development. To confirm the hypothesis, NUSAP1 was successfully silencing by ShNUSAP1 and recovered after transfected by lentiviruses in MEC-1 and EHEB cells (Fig. 3A). The absorbance values of cells were detected to show that NUSAP1 knockdown inhibited cell proliferation by CCK-8 indirectly (Fig. 3B). Flow cytometry was used to detect cell apoptosis. Down-regulating NUSAP1, the proportion of apoptotic cells was increased and then reverted after upregulating expression on basis of NUSAP1 knockdown (Fig. 3C, D). In addition, after silencing NUSAP1, MEC-1 and EHEB cells were blocked in G0/G1 phase. While recovering the expression of NUSAP1, G0/G1 phase fell accordingly (Fig. 3E, F).

### NUSAP1 knockdown induced the activation of DNA damage in CLL cells

**Fig. 4:**
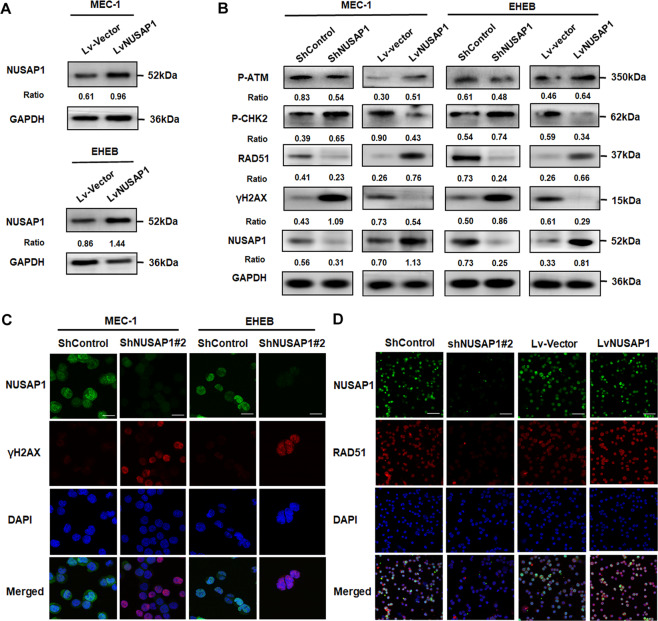
DNA damage in CLL cells induced by suppression of NUSAP1. **A** Western blotting verification of CLL cells with NUSAP1 overexpression. **B** Protein expression of DDR in CLL cells with NUSAP1 decrement and overexpression by western blotting analysis. **C** IF staining of γH2AX in CLL cells with NUSAP1-expression changed. Bar: 20 μm. **D** IF staining of RAD51 in MEC-1 cells with NUSAP1-expression changed. Bar: 50 μm.

With RNA-sequencing, it was discovered that NUSAP1 was notably related to the DNA damage repairing. With an assumption that NUSAP1 may serve as a regulatory factor in DDR, active forms of proteins engaged in DDR were inspected by western blotting. The protein expression levels of NUSAP1 were successfully rising after lentiviruses transfected (Fig. 4A). The results showed that in the NUSAP1 inhibition group, the levels of p-CHK2 and phosphorylated histone H2AX (γH2AX) were upregulated, while both the p-ATM and RAD51 appeared low expression (Fig. 4B). Among them, γH2AX is regarded as an indicator of DNA damage for recruiting into the lesions upon activation [24]. We further observed that the densities of γH2AX increased significantly after NUSAP1 knockdown by immunofluorescence (Fig. 4C), which means enhancing DNA damage activities while inhibiting NUSAP1 in CLL cells. As is shown in GSEA, NUSAP1 was related to homologous combination (HR). It was discovered that the protein levels of RAD51, an vital role in HR [25], were significantly reduced in NUSAP1 downregulated cells (Fig. 4B). Furthermore, RAD51 staining was decreased in cells with downregulated NUSAP1 expression, and elevated in cells with overexpression of NUSAP1 (Fig. 4D). However, no effect on RAD51 mRNA levels were observed after interference neither in CLL cell lines nor in primary cells of CLL patients (Supplementary Fig. 1). It was assumed that RAD51 was adjusted by NUSAP1 through post-translational modification (PTM).

### NUSAP1 bound with RAD51 through its C-terminus

**Fig. 5:**
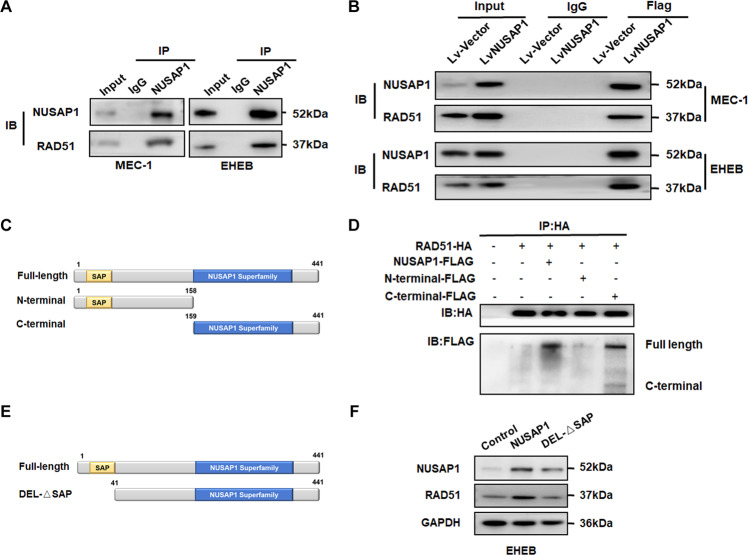
NUSAP1 bound with RAD51 via the C-terminus. **A** Endogenous interaction of NUSAP1 with RAD51 verification by Co-IP. **B** Exogenous interaction of NUSAP1 with RAD51 verification by Co-IP. **C** Structural representation of main domains of NUSAP1. **D** Interaction of RAD51 with truncated NUSAP1 in EHEB cells. **E** The SAP domain on the N-terminus of NUSAP1. **F** The protein expression levels of RAD51 while upregulating NUSAP1 and NUSAP1-ΔSAP in EHEB cells.

We hypothesized that NUSAP was bound with RAD51 and performed co-immunoprecipitation (CO-IP) assay. First, both endogenous and exogenous protein interactions between NUSAP1 and RAD51 were verified (Fig. 5A, B). In order to figure out which domain of NUSAP1 the RAD51 bound with, we transfected the different parts of plasmids into cells as shown in the Fig. 5C. In addition, NUSAP1 truncated vectors and full-length RAD51 were co-transfected into MEC-1 cells. The results demonstrated that RAD51 is bound with the C-terminal of NUSAP1 (Fig. 5D). Previous studies have displayed that NUSAP1 incorporates an SAP domain that is also involved in SUMO E3 ligase recognizing substrate [26]. It has been verified that SUMOylation could antagonize the independent degradation of ubiquitin [27]. In order to detect the role of the SAP domain of NUSAP1, NUSAP1-ΔSAP (amino acid 41-441) was constructed without the SAP domain (Fig. 5E). Compared with the NUSAP1 upregulated group, the NUSAP1-ΔSAP transfection group suppressed RAD51 expression as expected (Fig. 5F).

### NUSAP1 promotes chemoresistance in CLL by regulating RAD51

**Fig. 6:**
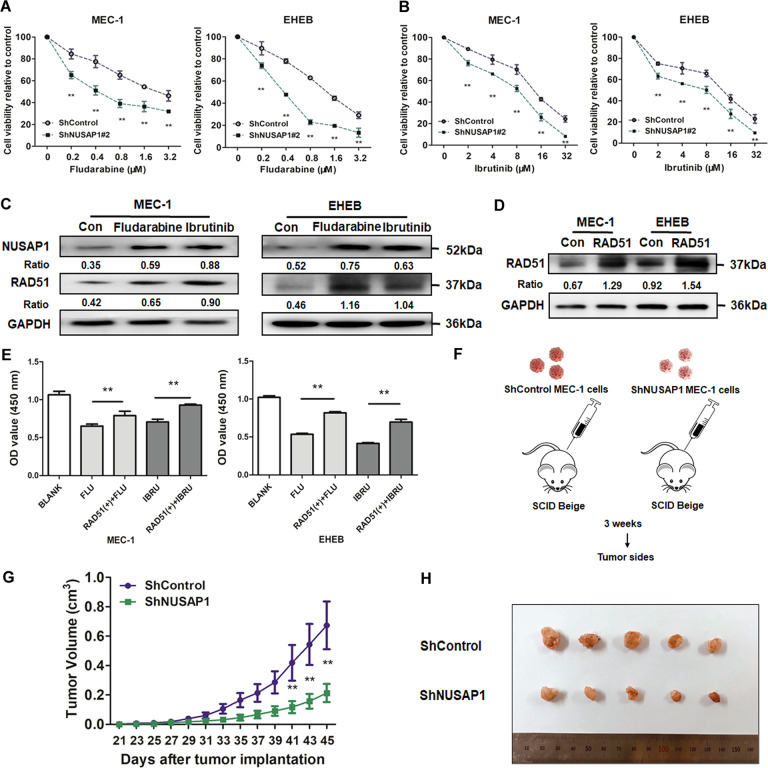
NUSAP1 reduction enhanced drug sensitivity for CLL cells and inhibited CLL cell growth in vivo. **A** The cell viability of NUSAP1 silencing cells under fludarabine for 48 h. **B** The cell viability of NUSAP1 silencing cells added with ibrutinib for 48 h. All data are presented as the mean ± SD, *n* = 3. *p < 0.05; **p < 0.01. **C** Changes of NUSAP1 and RAD51 before and after treatment of fludarabine and ibrutinib. **D** Transfection efficiency of RAD51 plasmid in CLL cells. (E) The OD value of CLL cells with RAD51 changed under treatment of fludarabine and ibrutinib. All data are presented as the mean ± SD, *n* = 3. *p < 0.05; **p < 0.01. **F** MEC-1 cells were implanted subcutaneously into the right inferior legs of SCID Beige mice. **G** Tumor volumes were measured every 2 days. Data are shown as the mean ± SD, *n* = 5. *p < 0.05, **p < 0.01. (**H**) After 45 days, obvious decreased tumor sizes were observed between ShControl and ShNUSAP1 group.

Regarded as a Bruton’s tyrosine kinase (BTK) inhibitor, ibrutinib served as the primary treatment for CLL. To verify whether NUSAP1 takes part in the adjustment of chemotherapy resistance, we addressed CLL cells with fludarabine and ibrutinib. Both the fludarabine and ibrutinib concentration gradients were referred to in previous research about CLL [28]. After NUSAP1 knockdown, both MEC-1 and EHEB cells treated with fludarabine and ibrutinib exhibited lower cell viability (Fig. 6A, B). RAD51 acts as a crucial feature in DNA damage repair and chemotherapeutic resistance. Besides, the protein expression levels of NUSAP1 and RAD51 elevated under drugs treatment (Fig. 6C), which implied possible mechanisms in chemoresistance. Then RAD51 plasmids were transfected to explore its function of this process (Fig. 6D). The cells viabilities in groups with both drugs interference and RAD51 overexpression were higher than drug intervention groups in 48 h (Fig. 6E). These results implied that RAD51 could engaged in chemoresistance.

### Suppression of NUSAP1 in CLL inhibits tumorigenesis in vivo

To further investigate whether NUSAP1 impacts on the growth of CLL cells in vivo, we observed the effects of NUSAP1 on tumor formation in SCID Beige implantation models. A total of 1 ×10^7^ ShControl and ShNUSAP1 MEC-1 cells resuspended in 100 μl PBS were subcutaneously injected into (SCID) beige mice (*n* = 5 per group) respectively as previously described [29] (Fig. 6F). Tumor sizes were measured every 2 days. After 45 days, tumor volumes were significantly reduced in mice treated with ShNUSAP1 cells compared to ShControl Group (Fig. 6G). It was proved that NUSAP1 reduction noticeably restricted tumor formation in vivo (Fig. 6H).

## Discussion

This study elucidated the carcinogenic role and regulatory mechanism of NUSAP1 for the first time in CLL development. Our evidences identified that NUSAP1 was elevated in CLL cells and specimens, which was related to poor prognosis of CLL patients. The suppression of NUSAP1 reduced the growth of CLL cells both in vivo and vitro, which also enhanced the sensitivity of CLL cells to fludarabine and ibrutinib. NUSAP1 exhibited an oncogenic role of triggering the DNA damage repair pathway by promoting downstream RAD51 expression.

As a prognostic factor, NUSAP1 is abnormally upregulated in some cancers [13, 14, 30]. But it has never been found in CLL. Consistently, we observed a significant increasement the of NUSAP1 expression in CLL cells and patient specimens relative to normal CD19 + B cells. Furthermore, high NUSAP1 expression was associated with poor survival time of CLL patients, as shown in GSE22762. However, more enrolled patients were needed for further studies to confirm the prognostic value of NUSAP1 in CLL. In order to investigate the molecular function and mechanism of NUSAP1 in CLL progression, we analyzed the role of NUSAP1 in CLL cells by RNA sequencing and vitro experiments. Via changing bidirectional expression of NUSAP1 targeting MEC-1 and EHEB cells, our results showed that inhibition of NUSAP1 induced cell proliferation decline, apoptosis enhancement, and G0/G1 cell cycle arrest. Recent evidences suggested that silencing NUSAP1 inhibits proliferation, migration, and invasion of glioma [31], breast cancer [32], liver cancer [6], and colorectal cancer [33]. However, its role in cell cycle progression remained controversial. NUSAP1 knockdown induced G0/G1 phase arrest in gastric cancer (GC) [30] and hepatocellular carcinoma (HC) cells [13]. Conversely, it was also discovered that silence of NUSAP1 resulted in G2/M arrest of renal cell carcinoma (RCC) cells. In addition, previous studies have shown that NUSAP1 mainly aggregates in pathways such as BTG2/PI3K/Akt signaling [14], Wnt/β-catenin signaling [34], mTORC1 signaling [30], and Hedgehog signaling pathway [13]. Similarly, KEGG and GSEA analysis suggested that NUSAP1 might promote CLL by regulating several oncogenic signaling pathways, especially in DNA repair pathway. In conclusion, we provided evidences that NUSAP1 might act as an oncogene in CLL, and further studies would describe the regulatory role of NUSAP1 in the DNA repair process.

The capacity of tumor cells to repair DNA damage induced by radiotherapy or chemotherapy also serves as one of the mechanisms of drug resistance [35]. As the most significant type of DNA damage, DNA double-strand breaks (DSBs) activate two important repair pathways, homologous recombination (HR) or non-homologous terminal connection pathway (NHEJ), accompanied with activating corresponding ATM/CHK2 or ATR/CHK1 signal pathways for repair [36]. In CLL, the ATM-CHK2-p53 signaling pathway in DNA damage response is frequently mutated and abnormally regulated [37].γH2AX and CHK2 phosphorylation, generally used as markers of DNA damage, are essential process associated with DSB [38, 39]. Consistently, increased levels of γH2AX and p-CHK2 were detected in NUSAP1 silencing CLL cells, indicating a stimulation of DNA damage [40] mediated by active ATM [41]. Whereas, the exact mechanism by which NUSAP1 acts on the DNA damage repair pathway needed further research. Notably, RNA-seq analysis was performed to investigate that NUSAP1 was mainly related to HR, of which RAD51 recruited by ATM to complete repair [42].

**Fig. 7:**
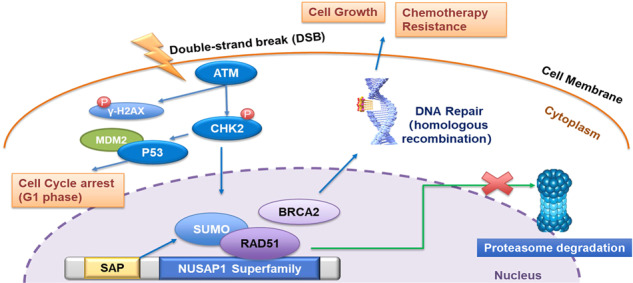
Diagram of the mechanism of NUSAP1 promoting DNA repair process by binding with RAD51. RAD51 bound with the C-terminus of NUSAP1, meanwhile the SAP domain at the N-terminus of NUSAP1 may promote SUMOylation of RAD51, which prevented it from degradation, contributing to DNA damage repair pathway and promoting cancer cell growth or enhancing therapy resistance.

Thus far, RAD51 is regarded as a desirable target in cancer therapy [43, 44]. In addition, the expression of RAD51 was decreased under silencing of NUSAP1 and enhanced along with upregulation of NUSAP1 in CLL cells, which illustrating that NUSAP1 has a positive effect on the RAD51 expression. We assumed that NUSAP1 might induce DNA repair process through binding with RAD51, which may prevent DNA damage from inhibiting the growth of CLL cells. Intriguingly, ubiquitination and SUMOylation modify proteins through PTM by attaching to lysine of indicator substrate. Different from ubiquitination, which usually degrades a specified substrate through a pathway rely on proteasome [45], SUMOylation stabilize proteins [46]. An obvious SAP domain, reported to recognize substrate and act ligase [26], was placed in the N-terminal of NUSAP1 [47]. Our results demonstrated that RAD51 bound with the C-terminus of NUSAP1, while the SAP domain at NUSAP1 N-terminus might facilitate the prime modeling of RAD51 (Fig. 7) and prevent the degradation of RAD51 through SAP domain. However, the SUMOylation process needs to be further researched.

Recently, remarkable efficacy has been displayed in CLL treatment by using target therapies alone or along with DNA damage drugs [48]. Fludarabine [49], one of the DNA synthesis inhibitor, is frequently used in CLL treatment regimen. Ibrutinib, irreversible Bruton’s tyrosine kinase inhibitors (BTKi), also emerges remarkable effects as therapy agents for both previously untreated and relapsed/refractory CLL patients [50]. However, the drug resistance and side effects emerged in clinical therapy are still challenges to be solved [51]. To explore the role of NUSAP1 in the chemotherapy resistance, we measured CLL cells viability with the change of NUSAP1. As supposed, inhibiting NUSAP1 elevated the sensitivity of CLL cells to fludarabine and ibrutinib treatment. The B-cell receptor (BCR) signaling pathway is aberrantly activated in the CLL, which makes BTKi an effective way for CLL patients’ treatment [52]. Activation of PI3K is associated with the conversion of phosphatidylinositol 4,5-bisphosphate (PIP2) to phosphatidylinositol 1,4,5-trisphosphate (PIP3), which assists BTK phosphorylation [53]. It is indicated that NUSAP1 knockdown inhibited cell growth and metastasis via regulating BTG2/PI3K/AKT signaling in nonsmall-cell lung cancer [14]. The suppression of NUSAP1 decreased the expression levels of p-PI3K and p-AKT to inhibit BTK phosphorylation. This BCR signalosome generates a wide variety of downstream effects, including activation downstream PLC2, ERK1/2, JNK, and AKT, thus modulating the migration and adhesion of B cells [54], which explained why NUSAP1 knockdown enhanced sensitivity to ibrutinib in our study. Fludarabine, as a DNA damaging agent in the standard regimen for CLL patients [55], was relevant to ATM/CHK2/γH2AX pathway [56], which NUSAP1 engaged in. Previous research indicated MEC-1 cells were more sensitivity treated with RAD51 inhibitor than fludarabine [56]. Based on overexpression level of RAD51 linked to chemoresistance [57] and poor outcome [19], the relationship between fludarabine resistance and RAD51 expression was further studied. Upregulated RAD51, consistently, the sensitivity of cells to fludarabine and ibrutinib decreased. Therefore, NUSAP1 enhances drug resistance by binding to RAD51 through DNA damage repair pathway in CLL.

In conclusion, we propose that the NUSAP1 contributes to DNA damage repairing and promoting stable proliferation in CLL for the first time. NUSAP1 suppression reinforces the response sensitivity of CLL cells to chemotherapy through attenuating RAD51 expression. This research suggests that NUSAP1 acts as a novel potential target in CLL treatment. Taken together, considering that NUSAP1 takes part in chemoresistance, an encouraging therapeutic strategy, which combines with NUSAP1 inhibition could be developed.

## Materials and Methods

### Cell culture

The human CLL cell line, EHEB, was derived from American Type Culture Collection (ATCC, Manassas, VA, USA). The cell line with p53 deletion/mutation, MEC-1, came from Professor Liguang Chen of Morse Cancer Center, University of California, San Diego, USA. The U87, HELA, and SKOV3 cell lines came from Shuai Ren of Shandong Provincial Hospital, Shandong, China. These types of cells were cultured in IMDM, RPMI-1640 and DMEM medium added 10% fetal bovine serum (Gibco, MD, USA), and placed in an incubator atmosphere of 5% CO_2_ at 37 °C. All of the cells have been regularly tested for mycoplasma infection.

### Silico analysis and RNA-seq

The gene symbols were annotated from the gene-expression omnibus (GEO) database GSE22762 through the R package illumine HumanWGDASLv3.db data probe. Then the survival of patients under differential gene expression was analyzed by the Limma package. Total cell RNA was extracted with RNAiso Plus (TaKaRa, Dalian, China). 3 stable ShNUSAP1 and 3 ShControl transfected MEC-1 cell samples were carried out for analysis by Huada Gene Technology Co. Ltd (Shenzhen, China) on basis of Illumina HiSeq 4000 platform.

### Patient specimens

This study was approved by the Shandong Provincial Hospital Affiliated to Shandong University Medical Ethics Committee with the informed consent of each patient based on the Helsinki Declaration. 51 CLL patients newly diagnosed according to the International Workshop on Chronic Lymphocytic revised criterion were collected from January 2013 to September 2019. All samples were obtained following informed consent. The peripheral blood mononuclear cells of patients were extracted by Ficoll-Hypaque density gradient (TBD Science, Tianjin, China). And normal CD19 + B cells were extracted by CD19 + magnetic microbeads kit (Miltenyi Biotec, Bergisch Gladbach, Germany) according to the method previously reported [12, 28].

### RNA isolation and quantitative real-time PCR

Total RNA was purified by using RNAiso Plus (TaKaRa, Dalian, China). RNA concentration was measured via NanoDrop 2000 spectrophotometer (Thermo Fisher Scientific, WALTHAM, MA). Reverse transcription was taken by high-volume complementary DNA reverse transcription kit (TaKaRa, Dalian, China). Quantitative real-time polymerase chain reaction (qRT-PCR) was conducted through the Power SYBR™ Green PCR Master Mix (TaKaRa, Dalian, China) on a 7400 real-time PCR system based on the manufacturer’s instructions. Finally, the fold change representing the mRNA expression levels of genes was calculated in terms of 2^− ΔΔCT^. GAPDH served as an internal reference. Primer sequences were as follows: GAPDH-F: 5′-GCACCGTCAAGGCTGAGAAC-3′; GAPDH-R: 5′-TGGTGAAGACGCCAGTGGA-3′; NUSAP1-F: 5′-CCATCTTCCGAGTATCGCCG-3′; NUSAP1-R: 5′-CAACTTGGTTGCCCTCAGGT -3′.

### Western blotting

The cells protein was isolated by dissolving buffer solution with protease inhibitor (Beyotime, Shanghai, China) and placed on ice for 0.5 h. The bicinchoninic acid assay (BCA) method was put to measure protein concentration after centrifugation (Shenergy Biocolor, Shanghai, China). Proteins in equal amounts were added into the electrophoresis on sodium dodecyl sulfate-polyacrylamide gel hole and separated at 200 V for 30 min. Then transfer the protein onto polyvinylidene difluoride filter membranes at 10 V for 30 min. Next seal the membranes in 5% fat-free milk at room temperature for 1 h, and incubated overnight with primary antibodies of NUSPA1, p-ATM, p-CHK2, γH2AX, p-BRCA1, RAD51 (Cell Signaling Technologies, Beverly, MA, USA), β-actin, and GAPDH (Zhongshan Goldenbridge, Beijing, China) at 4 °C. After washing three times in Tris-buffered saline containing Tween (TBST), the membranes were incubated at room temperature for 1 h with appropriate secondary antibody (Zhongshan Goldenbridge, Beijing, China). After another three times washing, the chemiluminescence reagent (Pierce) was covered in the membranes for visualization at Bio-Rad Image Lab ™ (Bio-Rad, Hercules, CA) and the protein gray density quantification was analyzed via the ImageJ software. β-actin and GAPDH served as an internal reference.

### Cell proliferation assays

Cell proliferation was detected with cell Counting Kit-8 (Beyotime, Shanghai, China) according to instruction. 10^4^ transfected cells/well were seeded into 96-well plates and then incubated for 24, 48, and 72 h. At the certain time, add 10 μl CCK-8 solution to each well and wait for another 2 h. The absorbance was measured at 450 nm with a the SpectraMax M2 Microplate Reader (Molecular Devices, CA, USA).

### Immunofluorescence assays

MEC-1 and EHEB cells were pressed on the glass slides, infiltrated in 4% paraformaldehyde for 15 min, and soaked with 0.1% Triton X 100 for 10 min. Next, the slides were blocked by 5% goat serum for 1 h, followed by incubation with antibodies at 4 °C overnight. After further incubation with Dylight 488-conjugated goat anti-rat IgG antibody (Abbkine, Beijing, China) for 1 h at room temperature, the slides were washed and stained by DAPI. Microanalysis was performed under a Nikon C2 confocal microscope.

### Analysis of cell apoptosis and cell cycle

Annexin V-PE/7AAD Kit (BD Biosciences, Bedford, MA, USA) was used for the analysis of cell apoptosis. After transfection, CLL cells were resuspended with Annexin V binding buffer. The cell suspension was added with Annexin V-PE/7AAD mix for 5 min. Apoptosis numbers was analyzed by Flowjo software. Cells for cell cycle analysis were collected in PBS, immersed with 70% ethanol at 4 °C overnight. Then stained with PI/RNase Staining Buffer (BD Biosciences, Bedford, MA, USA) for 15 min. Both types of assays were detected by Navios flow cytometer (Beckman Coulter Inc. USA).

### Lentivirus vector and plasmids transfection

The lentivirus vectors of ShNUSAP1 and control lentiviral vectors (Hu6-MCS-CBh-gcGFP-IRES-puromycin) were purchased from Genechem (Shanghai, China). The RNAi sequences were as follows: shNUSAP1#1, 5′-GGAAATGGAGTCCATTGATCA-3′; shNUSAP1#2, 5′-GCACCAAGAAGCTGAGAATGC-3′. The lentivirus vectors of LvNUSAP1 overexpressing NUSAP1 and Lv-empty vectors (Ubi-MCS-3FLAG-CBh-gcGFP-IRES-puromycin) were constructed by Genechem (Shanghai, China). The Infected with lentiviral vectors with MOI of 100, cell lines were placed in 96-well plates (10^4^ cells/well), and maintained with puromycin (1.0 μg/mL). After 72 h stable cells were cultured.

The full length, N-terminus, C-terminus of NUSAP1 plasmid, and the full lengths of RAD51 plasmid were conducted by Boshang (Jinan, Shandong). Lipo2000 (Invitrogen, California, USA) was used to transfect plasmid into cells. 1 μg plasmid was diluted with 50 μl opti-MEM for 5 min. 1 μl lipo2000 was diluted into 50 μl opti-MEM in 5 min. Then blend the diluted plasmid and lipo2000 gently. After 25 min, the mixture was added into cell culture. The transfected cells were collected 48 h later for drug treatment or western blot.

### Co-Immunoprecipitation (Co-IP) assay

The cells were collected and dissolved with Co-IP lysis buffer. After centrifuged, the supernatant lysis buffer was added with 1–3 ug primary antibody, shaken, and incubated at 4 °C overnight. The next day, protein A/G PLUS Agarose beads (Santa Cruz Biotechnology, USA) were added into the buffer to bind with antibodies, shaken, and incubated at 4 °C for 2 h. The beads were washed in PBS for 3 times and heated at 100 °C for denaturation. Then western blotting was used to detect the protein.

### Xenograft in vivo

A total of 1 × 10^7^ ShControl and ShNUSAP1 transfected MEC-1 cells resuspended in 100 μl PBS were respectively implanted into the female severe combined immunodeficiency (SCID) beige mice (*n* = 5 per group, 4-week-old) subcutaneously. All mice used in this study were chosen randomly. Experiments were not blinded. Tumor sizes of SCID beige mice were observed and measured every 2 days. The volume calculating equation was: V = (l × w^2^) ×0.5, where l was the largest diameter and w was the vertical dimension. All animal experiments were carried out based on the procedures approved by Institutional Animal Care and Research Advisory Committee of Shandong Provincial Hospital Affiliated to Shandong University (SPHASU).

### Statistical analysis

All the data in this paper were statistically analyzed by SPSS 17.0 software (Chicago, IL, USA) and Graphpad Prism 7.0 statistical software (San Diego, CA, USA). The results from 3 separate experiments were presented as mean ± standard deviation (SD). Student’s t test and Mann–Whitney *U* test were performed for direct comparisons. One-way ANOVA or two-way ANOVA were utilized for multigroup comparisons. The survival analysis was performed by the Kaplan–Meier method. *p < 0.05 was regarded as statistically significant. Correlation between NUSAP1 expression and characteristics of CLL patients was determined through the two-tailed *χ*^2^ test or Fisher’s exact test.

## Supplementary information

Figure S1

Supplementary figure legend

## Data Availability

The datasets generated during the current study are available in the Gene Expression Omnibus repository with the accession number GSE185467.
