# Supplementary figures and images for: Nucleolar and spindle associated protein 1 enhances chemoresistance through DNA damage repair pathway in chronic lymphocytic leukemia by binding with RAD51
Source: Cell Death Dis. 2021 Nov 15;12(11):1083. doi: 10.1038/s41419-021-04368-2 (PMC8593035; doi:10.1038/s41419-021-04368-2)

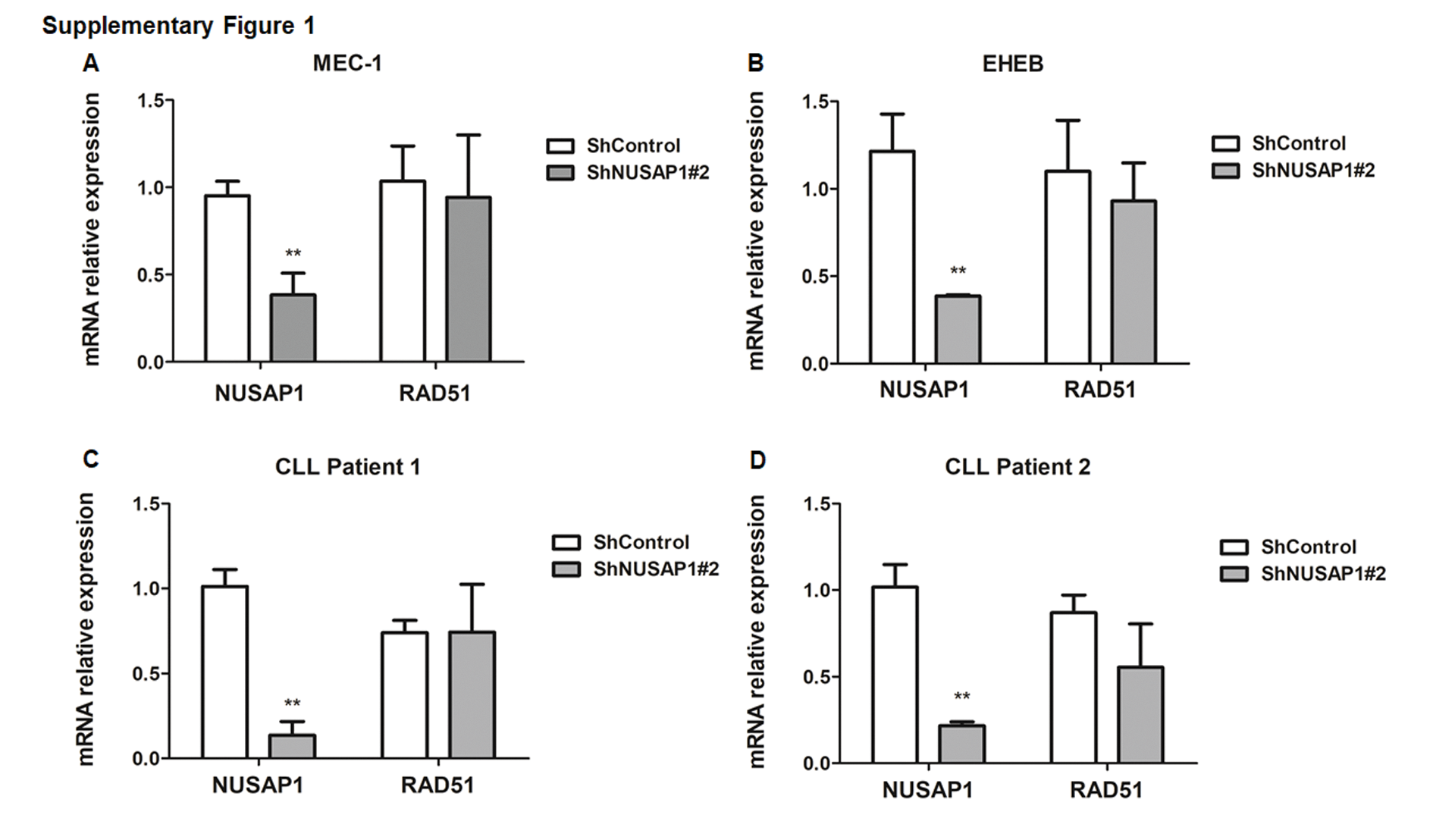

Supplement: Supplementary file 1 — Figure S1 [file 41419_2021_4368_MOESM1_ESM.tif]
