# Supplementary material for: Nucleolar and spindle associated protein 1 enhances chemoresistance through DNA damage repair pathway in chronic lymphocytic leukemia by binding with RAD51
Source: Cell Death Dis. 2021 Nov 15;12(11):1083. doi: 10.1038/s41419-021-04368-2 (PMC8593035; doi:10.1038/s41419-021-04368-2)
Supplement: Supplementary file 2 — Supplementary figure legend [file 41419_2021_4368_MOESM2_ESM.doc]

**Supplementary Figure legends**

**Supplementary Figure S1: The** [**transcriptional**](javascript:;) **levels variation of RAD51 accompanied with NUSAP1 silencing.** (A-B) The mRNA expression levels of NUSAP1 and RAD51 were tested between Shcontrol and ShNUSAP1#2 groups in MEC-1 and EHEB cells by qRT-PCR. (C-D) PBMCs were extracted from 2 CLL patients. After inhibiting NUSAP1 in CLL primary cells, the mRNA expression levels of NUSAP1 and RAD51 were detected by qRT-PCR. All data are presented as the mean ± SD, n = 3. **p* < 0.05; ***p* < 0.01.
